# Prediction of recidivism in a long-term follow-up of forensic psychiatric patients: Incremental effects of neuroimaging data

**DOI:** 10.1371/journal.pone.0217127

**Published:** 2019-05-16

**Authors:** Carl Delfin, Hedvig Krona, Peter Andiné, Erik Ryding, Märta Wallinius, Björn Hofvander

**Affiliations:** 1 Centre for Ethics, Law and Mental Health, Department of Psychiatry and Neurochemistry, Institute of Neuroscience and Physiology, The Sahlgrenska Academy at University of Gothenburg, Gothenburg, Sweden; 2 Regional Forensic Psychiatric Clinic, Växjö, Sweden; 3 Lund University, Faculty of Medicine, Department of Clinical Sciences Lund, Child and Adolescent Psychiatry, Lund, Sweden; 4 Forensic Psychiatric Clinic, Sahlgrenska University Hospital, Gothenburg, Sweden; 5 Department of Forensic Psychiatry, National Board of Forensic Medicine, Gothenburg, Sweden; 6 Department of Clinical Neurophysiology, Skåne University Hospital, Lund, Sweden; 7 Division of Forensic Psychiatry, Region Skåne, Trelleborg, Sweden; Medical University of Vienna, AUSTRIA

## Abstract

One of the primary objectives in forensic psychiatry, distinguishing it from other psychiatric disciplines, is risk management. Assessments of the risk of criminal recidivism are performed on a routine basis, as a baseline for risk management for populations involved in the criminal justice system. However, the risk assessment tools available to clinical practice are limited in their ability to predict recidivism. Recently, the prospect of incorporating neuroimaging data to improve the prediction of criminal behavior has received increased attention. In this study we investigated the feasibility of including neuroimaging data in the prediction of recidivism by studying whether the inclusion of resting-state regional cerebral blood flow measurements leads to an incremental increase in predictive performance over traditional risk factors. A subsample (*N* = 44) from a cohort of forensic psychiatric patients who underwent single-photon emission computed tomography neuroimaging and clinical psychiatric assessment during their court-ordered forensic psychiatric investigation were included in a long-term (ten year average time at risk) follow-up. A Baseline model with eight empirically established risk factors, and an Extended model which also included resting-state regional cerebral blood flow measurements from eight brain regions were estimated using random forest classification and compared using several predictive performance metrics. Including neuroimaging data in the Extended model increased the area under the receiver operating characteristic curve (AUC) from .69 to .81, increased accuracy from .64 to .82 and increased the scaled Brier score from .08 to .25, supporting the feasibility of including neuroimaging data in the prediction of recidivism in forensic psychiatric patients. Although our results hint at potential benefits in the domain of risk assessment, several limitations and ethical challenges are discussed. Further studies with larger, carefully characterized clinical samples utilizing higher-resolution neuroimaging techniques are warranted.

## Introduction

Crime in general and violent crime in particular is a significant public health concern. Decades of research have identified several risk factors for persistent criminality, and among the most widely reported are previous criminality, younger age, younger age at criminal onset, aggressive behavior, substance use and factors relating to dysfunction within family, school, and employment [1–9]. Furthermore, while persistent criminality is more common among males than females, risk factors appear to be similar between the sexes [10]. A comparable pattern emerges in individuals with mental disorders. Although previous research has found evidence of a substantially increased risk of violence in individuals with major mental disorders, even when adjusting for substance use and other known risk factors [11–13], a recent meta-analysis concluded that major mental disorders by themselves appear to be unreliable predictors of both general and violent recidivism [14]. Instead, risk factors in mentally disordered offenders seem to mirror those found in the general population [15,16], with the most prominent risk factors being the presence of an antisocial personality disorder and/or a high degree of psychopathic traits [14,17,18].

One of the primary objectives in forensic psychiatry is risk management. To that end, risk assessments are performed to predict criminal recidivism and provide a baseline for risk management, but the currently most used risk assessment tools–which in essence are made up of various constellations of the traditional risk factors outlined above–are limited in their ability to do just that [16,19,20]. With the limits of risk assessments based solely on traditional risk factors in mind, coupled with advances in neuroimaging and an emerging hypothesis that life-course persistent antisocial behavior may be viewed as a neurodevelopmental disorder [21], the prospect of incorporating neuroimaging data to improve the prediction of criminal behavior has received increased attention [16,19,20,22–24].

One of the most consistent neurobiological markers for antisocial behavior is reductions in frontal lobe structure and function, observed in several samples of criminal, violent and psychopathic individuals [25–28]. In addition, both structural and functional temporal lobe reductions have been reported in several studies [29–31], and reduced parietal glucose metabolism has been related to impulsive aggression, impulsive personality disorders, and violent offending [32–35]. Frontal and temporal aberrations are also consistently found in aggressive patients suffering from schizophrenia [36], and reductions in both frontal, temporal, parietal and cerebellar gray matter volume have been observed after the onset of psychosis [37]. More recently, aberrations in smaller regions such as the hippocampus [38], nucleus accumbens [39] and amygdala [40] have been observed in offenders with psychopathy, altered connectivity between the amygdala and the cerebellum has been found in violent offenders [41], and it is possible that the cerebellum itself may be related to psychopathic traits, recidivism and violent criminality [30,42].

Certainly, our understanding of the neurobiological underpinnings of criminal and violent behavior in mentally disordered individuals is far from conclusive, although the potential of utilizing neuroimaging data in the prediction of recidivism may hold some promise. To our knowledge only two studies, both using participants from the same sample of male prisoners, have been published where authors have incorporated neuroimaging data to predict recidivism [43,44]. In this retrospective and exploratory study we extend previous research into the domain of forensic psychiatry. We address the feasibility of including neuroimaging data in the prediction of recidivism by investigating if the prediction of recidivism using a Baseline model, with empirically well-established risk factors, could be improved by including resting-state regional cerebral blood flow (rCBF) measurements in an Extended model, in a long-term follow-up of forensic psychiatric patients.

## Materials and methods

### Participants

Participants (*N* = 44) were recruited from the Forensic psychiatric follow-up studies–the Malmö cohort (UPPRÄTT-Malmö study). The UPPRÄTT-Malmö study consists of 101 men and 24 women, aged 17–79 (median age = 38) at the time of inclusion. The UPPRÄTT-Malmö study is a nationally representative, total cohort of patients living in the Malmö University Hospital catchment area who, after committing a crime, underwent either a major forensic psychiatric investigation (FPI, *N* = 97) or a minor forensic psychiatric screening report (*N* = 28) between 1999 and 2005, and subsequently were sentenced to involuntary forensic psychiatric in-patient treatment. One previous study has investigated recidivism using traditional risk factors [45], and one previous study has investigated predictors of length of stay [46], both using the full UPPRÄTT-Malmö sample. When study inclusion commenced, FPI investigees were routinely being referred for a neuroimaging assessment, but due to changes in the local FPI procedures during study inclusion, only 50 participants underwent this assessment. Of those, six were omitted from the current study; two were missing data on educational attainment, three were missing data on age at first crime, and one was missing data on psychopathic traits due to lack of sufficient data for scoring. Thus, the final sample in this study was 44 participants, aged 20–79 (median age = 35).

### Clinical assessment and characteristics

Psychiatric diagnoses were assessed at the end of FPI according to the Diagnostic and Statistical Manual of Mental Disorders 4^th^ Edition (DSM-IV) [47] using semi-structured interviews by the Structured Clinical Interview for DSM-IV Axis I Disorders (SCID-I) [48] and the Structured Clinical Interview for DSM-IV Axis II Disorders (SCID-II) [49]. The diagnoses reflect the participant’s psychiatric status at the time of the FPI, and by definition, all individuals presented symptoms consistent with one or more major mental disorders at the time of both the FPI and the committed crime(s). We clustered participants’ primary DSM-IV diagnoses into five categories: psychotic disorder, mood disorder, personality disorder, cognitive disorders, and neurodevelopmental disorders. Information about participant’s age at the time of FPI was obtained from the FPI protocols, while dates of admittance (i.e., start of forensic psychiatric in-patient treatment) and discharge were obtained from patient records. The number of days under forensic psychiatric care was defined as the number of days between patient’s intake date and either date of discharge, date of death, date of deportation, or the 31st of December, 2013 (i.e., end of follow-up) if patients were still under forensic psychiatric care when follow-up ended.

### Neuroimaging data

The neuroimaging data used in this study was acquired using single-photon emission computed tomography (SPECT) and was collected as part of the FPI investigation. Thus, the choice of SPECT was clinically motivated rather than motivated by research. Measurements were carried out using ^99m^Tc-exametazime (Ceretec^TM^, Nycomed-Amersham/GE Healthcare) and a Ceraspect SPECT camera (Digital Scintigraphics Inc., Waltham, Massachusetts). Participants were administered 900 MBq of ^99m^Tc-exametazime through a pre-set cannula in a cubital vein while resting comfortably supine, awake and silent in a muted room with eyes open focusing on a point in the ceiling.

^99m^Tc-exametazime is lipophilic and passes through the blood-brain barrier and the cell membrane to reach intra-cellular space in proportion to rCBF. Intracellular ^99m^Tc-exametazime rapidly transforms into a polar form that cannot leave the cell, and thus the ^99m^Tc-exametazime distribution in the brain remains unchanged for several hours, providing a snapshot of rCBF a brief period after the injection. The imaging procedure began about 15 minutes after administration and participants were recorded for 30 minutes.

The radiation from the ^99m^Tc-exametazime was recorded in 180^0^ to allow a 3-dimensional reconstruction of the activity, proportional to the rCBF, after scatter and attenuation corrections, with a resolution of 9 mm FWHM (full-width at half-maximum). The recorded three-dimensional activity was saved into a 128*128*64 voxel matrix and subdivided into 10 slices with 1 cm thickness, parallel to the orbitomeatal line. A region-of-interest (ROI) set [50] was scaled to fit the outer dimensions of the brain for three dimensional measurement of activity, proportional to rCBF. The measured value in each ROI was quantified, using Amersham ROI software (GE Healthcare, Buckinghamshire, UK), in percent of the mean ^99m^Tc-exametazime concentration in the whole brain.

### Pharmacological treatment at the time of SPECT acquisition

A majority of Swedish forensic psychiatric patients receive pharmacological treatment, often with multiple agents, although antipsychotics are the most prevalent [51]. Antipsychotics are known to affect rCBF primarily in frontal, temporal and striatal regions [52], while anticholinergics, often administered to reduce extrapyramidal symptoms of antipsychotics, appear to reduce rCBF in the whole brain [53]. We collected data on pharmacological treatment from medical records from the time of SPECT acquisition and structured the data according to five major pharmacological categories: antipsychotics, antidepressants, benzodiazepine sedatives/hypnotics, non-benzodiazepine sedatives/hypnotics, and anticholinergics, each coded as either ‘yes’ or ‘no’.

### Follow-up data on criminality

To account for the fact that patients in some cases relapse in crime during their forensic psychiatric care [54], the time at risk was defined as beginning at each patient’s intake date and lasting until reconviction, death, deportation or until the end of follow-up at the 31st of December 2013. Recidivism was defined as a criminal conviction during the time at risk and is presented as general recidivism (i.e. all convictions, including violent) due to low base rates of specific crimes. The mean time at risk for the entire sample was 3623 days (SD = 1495), ranging from 166 to 5342 days. Nine patients in the sample died during the follow-up, and had an average time at risk of 1477 days (SD = 1165), ranging from 166 to 3410 days. In addition, one patient was deported during the follow-up, and had a time at risk of 219 days. Dates of new crimes and convictions, dates of legal force of new sentences and following periods of sanctions, as well as dates of deportations were provided by the National Council of Crime Prevention. Dates of deaths were obtained from the Cause of Death Register at the National Board of Health and Welfare.

### Baseline model measures

Nine traditional risk factors were selected based on previous literature [1–6,14,45] to be included in the Baseline model: age at FPI, age at first crime, degree of psychopathic traits, sex, substance use disorder, cluster B personality disorder, educational attainment, mental disorder in first-degree relative, and previous criminality. Note that we opted to include both age at FPI and age at first crime as baseline risk factors not only because both are associated with recidivism, but also because prior research suggests that both global and regional CBF tends to decrease with age [55–61].

Information about participant’s sex, previous criminality (defined as any previous conviction *prior* to the crime that lead to FPI and dichotomized as ‘yes’ or ‘no’), age at first crime, educational attainment (dichotomized as ‘yes’ or ‘no’, with ‘yes’ indicating primary school or higher attainment), and mental disorder in first-degree relative (dichotomized as ‘yes’ or ‘no’) was obtained from the FPIs using structured protocols. In most instances, psychopathic traits were scored during the FPI based on information from the clinical assessment as well as extensive file and register reviews, using the Psychopathy Checklist: Screening Version (PCL-SV) [62], consisting of 12 items scored on a 3-point scale (0, 1, 2). When an item of the PCL:SV was omitted, a score was assigned according to the PCL:SV manual. In cases where PCL:SV ratings were missing (*N* = 5 of the current study sample), these were performed retrospectively, based on file reviews [63].

### Extended model measures

**Fig 1:**
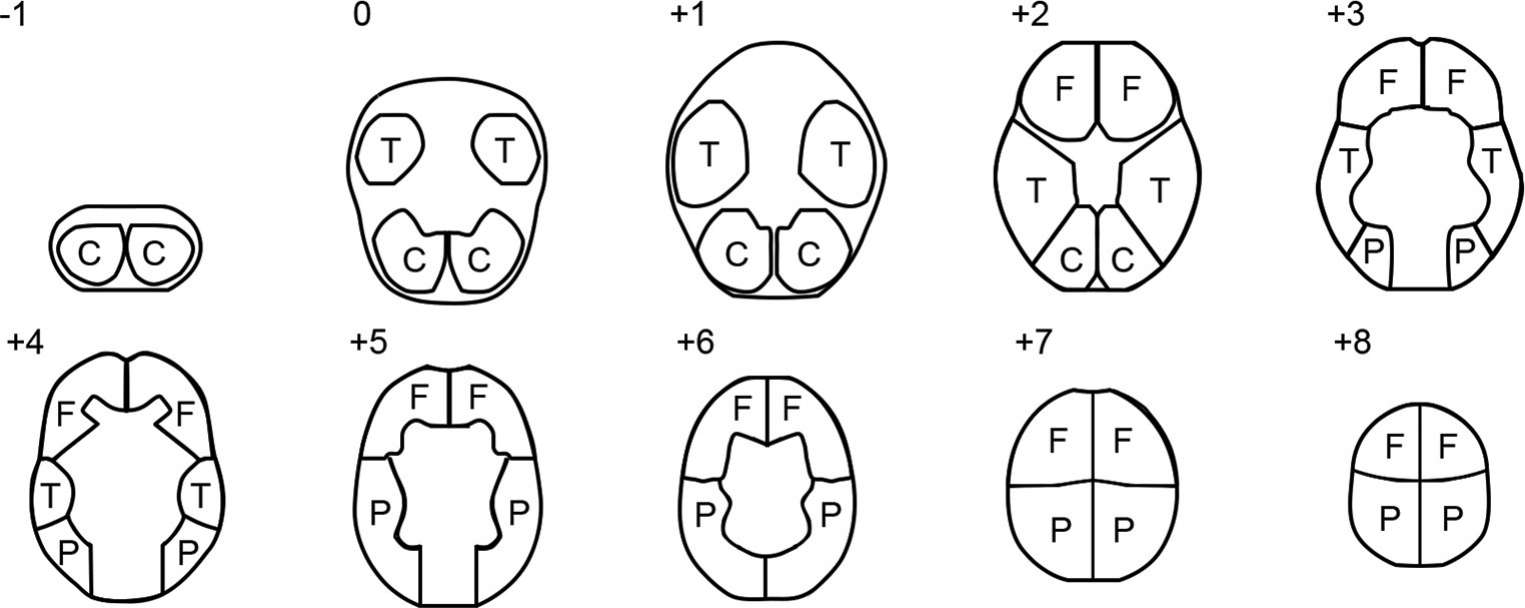
Regions-of-interest. An overview of the regions-of-interest (ROIs) used in the current study. Numbers refer to centimeters above/below the orbitomeatal line. C = cerebellum, T = temporal lobe, F = frontal lobe, P = parietal lobe.

The Extended model consisted of the variables used in the Baseline model plus neuroimaging data in the form of resting-state rCBF measurements. Given the exploratory nature of this study, and with a parsimonious approach to the number of predictors included, eight ROIs were selected (the left and right frontal lobe, the left and right parietal lobe, the left and right temporal lobe, and the left and right cerebellum; see Fig 1) ensuring coverage of the brain’s major regions (the occipital lobe was excluded since visual stimulation was part of the procedure to ensure participant’s wakefulness, and smaller volume regions such as the thalamus and basal ganglia were excluded due to the procedure’s low spatial resolution).

### Data analyses

Data preparation and statistical analysis was conducted using the R statistical programming language [64]. All R code is publicly available at the corresponding authors’ GitHub page (https://github.com/carldelfin/neuroprediction), adhering to the principles of reproducible research [65]. Statistical significance was pre-defined as *p* < .05.

#### Group comparisons and correlations

Differences between recidivists and non-recidivists were examined using Barnard’s test [66] for dichotomous variables and Welch’s *t*-test for numerical variables. Barnard’s test is a more powerful alternative to Fisher’s exact test when sample sizes are small [67]. Welch’s *t*-test performs better than Student’s t-test in situations where sample size and variance is unequal between groups and equal to Student’s *t*-test in situations where sample size and variance is equal [68]. Correlations were examined using Spearman’s *ρ*.

#### Random forest classification

Random forest classification (RF) [69], a so-called ensemble method which aggregates the results from a large collection of decision trees [70], was used to predict recidivism. Recognized for its high accuracy, the RF machine learning algorithm has no distributional assumptions, performs well with both small sample sizes and high-dimensional data [71], and has previously been successfully used to predict general recidivism in mentally disordered offenders [72]. The RF algorithm works by building each decision tree using a random bootstrap sample (with replacement) corresponding to roughly 66% of the data. The remaining so-called out-of-bag (OOB) data is used for estimating model error and assessing variable importance, allowing the RF algorithm to reduce overfitting; an otherwise common occurrence in predictive modeling [73]. In addition, only a random subset of predictors is selected at each node in the decision tree. Each predictor is split to optimize tree performance, and the predictor split that produces the highest tree performance is selected for that node. After constructing a tree, each observation in the OOB data is passed down the tree and is classified, in the case of the present study, as either “yes” or “no” for recidivism. The final (i.e., aggregated) classification of each observation is the majority “vote” based on all the trees where that observation was in the out-of-bag sample.

Each model was created using 10 000 trees, using the default of √*p* predictors at each node, with *p* being the total number of predictors available. Since the RF algorithm can be sensitive to class imbalance, the majority class (i.e., non-recidivists) was down-sampled to ensure that each bootstrap sample contained the same number of non-recidivists as recidivists [74].

### Assessing model performance

Predictive performance was assessed using several metrics. We report the area under the receiver operating characteristic curve (AUC), representing the probability (from 0 to 1) that a randomly selected recidivist will have been predicted by the model as having a higher probability of recidivism than a randomly selected non-recidivist. The AUC has been put forward as the recommended measure of predictive performance in forensic psychiatry [75], although opinions differ about its predictive interpretation. For instance, an AUC of .71 corresponds to Cohen’s *d* = .80, which is a large effect size [75]. Others have suggested that AUCs in risk assessments should be more conservatively interpreted, with AUCs between .60 and .70 having modest accuracy and AUCs between .80 and .90 having moderate accuracy [76]. We also report accuracy (the overall proportion of correct classifications), sensitivity (the proportion of recidivists correctly classified as such), specificity (the proportion of non-recidivists correctly classified as such), positive predictive value (PPV; the proportion of predicted recidivists that actually are recidivists), and negative predictive value (NPV; the proportion of predicted non-recidivists that actually are non-recidivists). In addition, we report scaled Brier scores, as recommended by recent research [77], calculated using the DescTools R package. The (unscaled) Brier score is defined as the squared difference between the actual binary outcome *Y* (0 or 1) and the predicted probability *p* (ranging from 0 to 1). By scaling the Brier score so that it no longer depends on the prevalence of *Y*, the resulting scaled Brier score ranges between 0 and 1. Similar to Pearson’s *R*^2^, a higher scaled Brier scores indicates better calibration of the predictive model [78]. The advantage of (whether scaled or unscaled) Brier scores is that the best score will be attained by the model that is able to predict as close to the true probabilities as possible.

#### Assessing variable importance

Individual variable importance is estimated by the RF algorithm during the OOB phase by randomly permuting each variable and recording how it affects classification accuracy. If a variable is important for classification then permutation should result in a large decrease in classification accuracy, whereas for unimportant variables, permutation should have little to no effect on classification accuracy. We report the scaled (mean divided by SD) mean decrease in accuracy for each variable, which is an estimate of the decrease in model accuracy, should that variable be omitted. In addition, partial dependence plots [79] visualize both the direction and size of effect (a wider range in the y-axis implies a larger effect) of each variable, after averaging out the effect of all other variables.

## Ethics

The study was register-based and retrospective; all clinical data (including SPECT measurements) was routinely collected as part of the FPI during the time study inclusion commenced. Thus, informed consent was not considered necessary, as it would not be possible to contact most participants due to the length of time that had passed after finishing treatment and because contact could pose a risk to vulnerable subjects with mental health and/or legal problems. All procedures used in this study were approved by the regional ethics review board in Lund (2007/64 and 2014/911).

## Results

### Recidivism

Sixteen patients (36% of the sample) were convicted of a crime during their time at risk. Most crimes were non-violent, such as theft, fraud, falsification of documents, driving under the influence of alcohol, and drug offences, although seven patients (16% of the sample) were convicted of violent crimes, including assault and battery, unlawful threat, and robbery. There was no difference in time at risk between recidivists and non-recidivists (Table 1).

**Table 1:** Detailed overview of sample clinical characteristics.

	All (*N* = 44)	Non-recidivists (*N* = 28)	Recidivists (*N* = 16)		
	Mean (± SD) or *N* (%)	Mean (± SD) or *N* (%)	Mean (± SD) or *N* (%)	*t* or *z*	*p*
**Demographics and clinical characteristics**					
Patients still under forensic psychiatric care^a^	13 (30%)	8 (29%)	5 (31%)	-0.19	0.898
Number of days under forensic psychiatric care	1746.77 (± 1639.04)	1765.93 (± 1676.93)	1713.25 (± 1624.04)	0.1	0.919
Time at risk (days)	3622.86 (± 1494.5)	3427 (± 1678)	3965.62 (± 1066.6)	-1.3	0.201
**Primary DSM-IV diagnosis**					
Psychotic disorder	30 (68%)	20 (71%)	10 (62%)	0.61	0.596
Mood disorder	5 (11%)	3 (11%)	2 (12%)	-0.18	0.967
Personality disorder	1 (2%)	0 (0%)	1 (6%)	-1.34	0.246
Cognitive disorder	3 (7%)	2 (7%)	1 (6%)	0.11	0.998
Neurodevelopmental disorder	5 (11%)	3 (11%)	2 (12%)	-0.18	0.967
**Pharmacological treatment at the time of SPECT**					
Antipsychotic	27 (61%)	19 (68%)	8 (50%)	1.17	0.261
Antidepressant	13 (30%)	6 (21%)	7 (44%)	-1.56	0.131
Benzodiazepine sedatives	20 (45%)	12 (43%)	8 (50%)	-0.46	0.657
Non-benzodiazepine sedatives	16 (36%)	10 (36%)	6 (38%)	-0.12	0.923
Anticholinergic	10 (23%)	9 (32%)	1 (6%)	1.97	0.052
**SPECT rCBF measurements**					
Frontal (right)	106.61 (± 4.06)	106.46 (± 4.52)	106.88 (± 3.22)	-0.35	0.728
Frontal (left)	106.64 (± 3.94)	106.82 (± 4.34)	106.31 (± 3.22)	0.44	0.66
Parietal (right)	104.82 (± 3.2)	106.11 (± 2.74)	102.56 (± 2.71)	4.16	< .001
Parietal (left)	103.45 (± 3.59)	104.18 (± 4.11)	102.19 (± 1.94)	2.17	0.036
Temporal (right)	102.57 (± 3.39)	102.07 (± 3.67)	103.44 (± 2.73)	-1.4	0.168
Temporal (left)	101.52 (± 2.57)	101.04 (± 2.85)	102.38 (± 1.75)	-1.93	0.06
Cerebellum (right)	119.64 (± 4.69)	120.68 (± 4.6)	117.81 (± 4.4)	2.04	0.049
Cerebellum (left)	119.82 (± 5.01)	120.68 (± 4.92)	118.31 (± 4.95)	1.53	0.136
**Baseline model variables**					
Age at forensic psychiatric investigation	37.84 (± 14.79)	42.29 (± 16.28)	30.06 (± 6.95)	3.46	0.001
Age at first crime	30.34 (± 14.09)	34.57 (± 15.69)	22.94 (± 5.81)	3.52	0.001
PCL:SV Total Score	10.3 (± 5.97)	9.25 (± 5.6)	12.12 (± 6.32)	-1.51	0.142
Male sex	39 (89%)	25 (89%)	14 (88%)	0.18	0.967
Substance use disorder	22 (50%)	12 (43%)	10 (62%)	-1.25	0.238
Cluster B personality disorder	7 (16%)	1 (4%)	6 (38%)	-2.96	0.008
Educational attainment	40 (91%)	26 (93%)	14 (88%)	0.59	0.732
Mental disorder in first-degree relative	13 (30%)	7 (25%)	6 (38%)	-0.87	0.459
Previous criminality	28 (64%)	17 (61%)	11 (69%)	-0.53	0.608

PCL:SV = Psychopathy Checklist: Screening Version.

^a^At the end of follow-up on 31st of December 2013.

### Clinical characteristics

Slightly less than one third of the sample were still under forensic psychiatric care at the end of the follow-up, with an average length of stay of almost 4.8 years, and no significant difference between recidivists and non-recidivists. The primary diagnosis of the majority of patients was a psychotic disorder, and no significant difference was found between recidivists and non-recidivists (Table 1). Comorbidity was relatively common, with 59% of patients diagnosed with two or more DSM-IV Axis I disorders (median = 2, range = 1 to 8). The median number of DSM-IV Axis II disorders was 0, ranging from 0 to 3. Most patients received antipsychotics at the time of SPECT acquisition, with no significant differences regarding pharmacological treatment between recidivist and non-recidivists. Anticholinergic treatment did appear less common among recidivists, although the difference did not reach statistical significance at the pre-defined level (Table 1).

### rCBF measurements

Recidivists had significantly lower bilateral parietal lobe and right cerebellar rCBF compared to non-recidivists. They also exhibited slightly higher temporal lobe rCBF than the non-recidivist group, but the difference was not statistically significant at the pre-defined level (Table 1).

### Baseline risk factors

Recidivists were significantly younger at the time of FPI, and also had a significantly younger age at their first crime. There was also a significantly higher frequency of cluster B personality disorders among the recidivists. There were no significant differences regarding PCL:SV total score, sex, substance use disorder, educational attainment, mental disorder in first-degree relative, or previous criminality (Table 1).

### Associations between age at FPI and rCBF

Age at FPI was significantly and positively associated with right parietal lobe rCBF (*ρ* = .31, *p* = .039) and significantly and negatively associated with left frontal lobe rCBF (*ρ* = -.35, *p* = .02). Associations between age at FPI and right cerebellar (*ρ* = .28, *p* = .065), left cerebellar (*ρ* = .27, *p* = .074), right frontal lobe (*ρ* = -.09, *p* = .555), right temporal lobe (*ρ* = .12, *p* = .456), left temporal lobe (*ρ* = -.23, *p* = .134), and left parietal lobe rCBF (*ρ* = .18, *p* = .237) did not reach the predetermined level of significance.

### Baseline model

**Fig 2:**
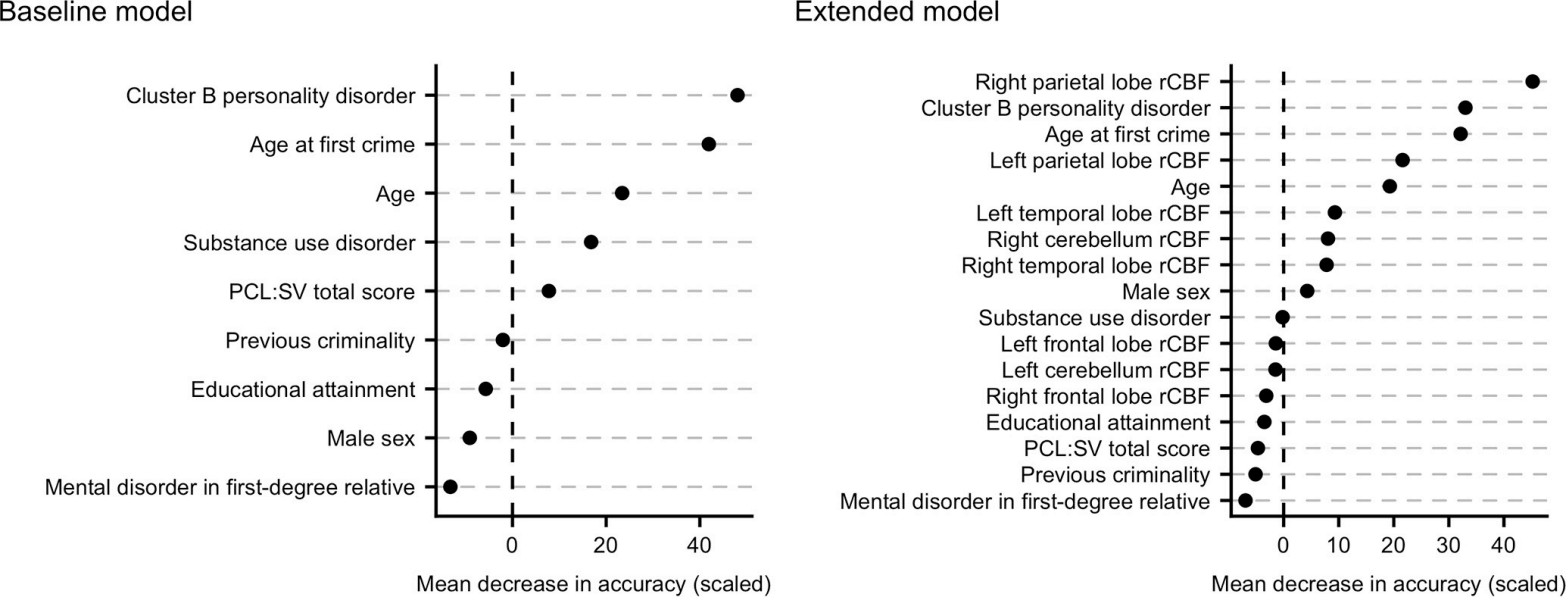
Variable importance. Variable importance measured as the scaled mean decrease in accuracy of each variable in the Baseline and Extended model. A higher value confers a higher decrease in the accuracy of the model, should that variable be omitted.

**Fig 3:**
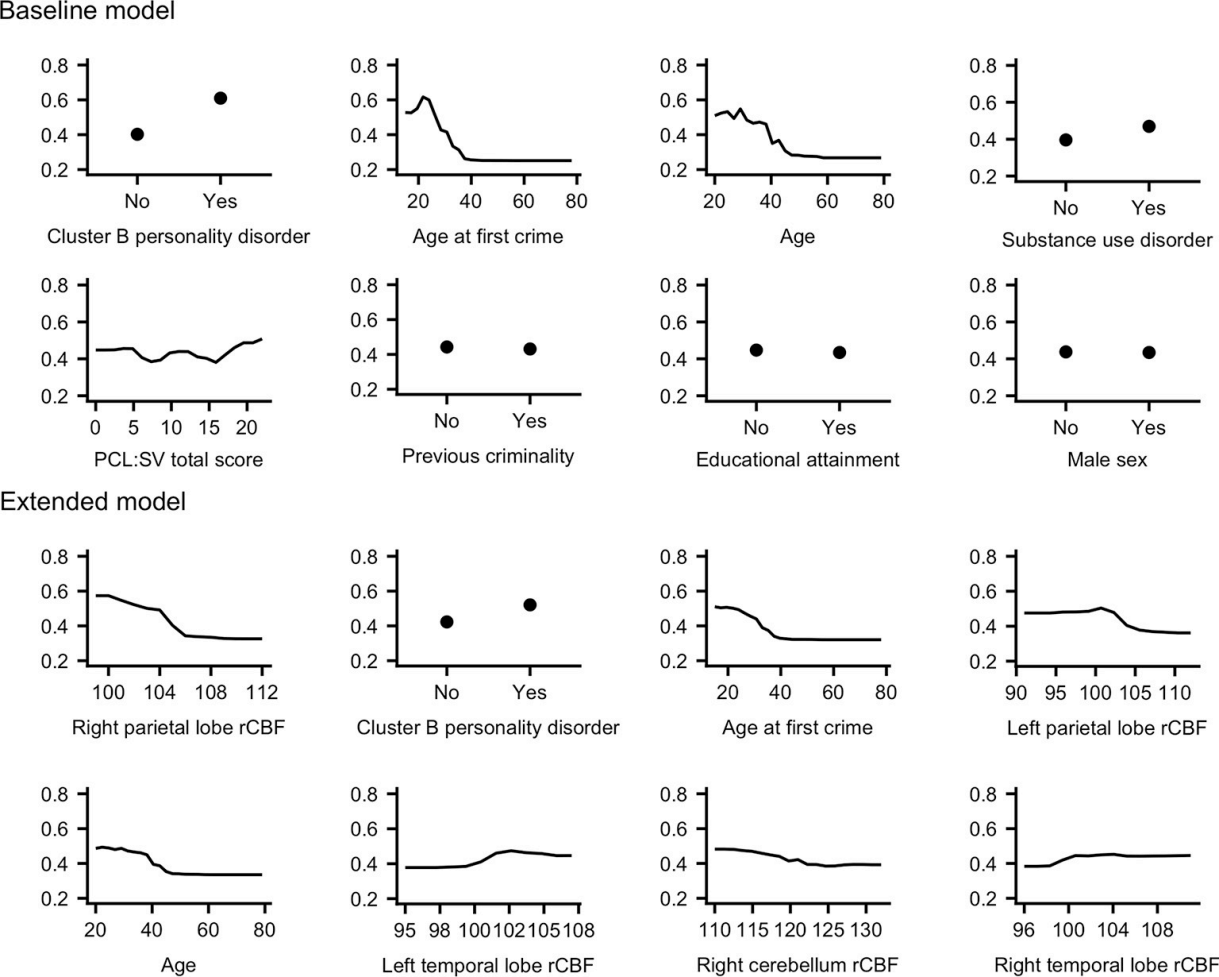
Partial dependence plots. Partial dependence plots for the eight most important (in terms of scaled mean decrease in accuracy) variables in each model. A higher value on the y-axis confers a higher probability of being predicted as a recidivist for the corresponding value on the x-axis for that variable.

Predictive performance of the Baseline model was modest, all measures considered. The AUC was .69, with a scaled Brier score of .08 and an accuracy of .64 (95% CI [.48, .78]), and sensitivity = .63, specificity = .64, PPV = .50, and NPV = .75. The most important variables in terms of mean decrease in accuracy were cluster B personality disorders, age at first crime, age, and substance use disorders (Fig 2, left panel). Partial dependence plots revealed that a cluster B personality disorder, lower age at first crime, younger age at the time of FPI, and substance use disorders increased the probability of being classified as a recidivist (Fig 3, top panel).

### Extended model

Predictive performance increased across all metrics in the Extended model. The AUC was .81 with a scaled Brier score of .25 and an accuracy of .82 (95% CI [.67, .92]), and sensitivity = .75, specificity = .86, PPV = .73, and NPV = .86. The most important variables were right parietal rCBF, cluster B personality disorder, age at first crime, and left parietal rCBF (Fig 2, right panel). In terms of actual predictions, the Extended model correctly classified two additional recidivists and six additional non-recidivists compared to the Baseline model, resulting in 12 out of 16 recidivists and 24 out of 28 non-recidivists being correctly classified by the Extended model. The probability of being classified as a recidivist increased with lower right parietal lobe rCBF, a cluster B personality disorder, lower age at first crime, and lower left parietal lobe rCBF (Fig 3, bottom panel). A similar effect was visible for lower right cerebellar rCBF and lower age, although less pronounced. Conversely, higher (bilateral) temporal lobe rCBF modestly increased the probability of being classified as a recidivist (Fig 3, bottom panel).

### Supplementary analysis of pharmacological data

Despite no statistically significant differences in pharmacological treatment between recidivists and non-recidivists, we conducted a supplementary analysis before ruling out any potential effects of pharmacological treatment at the time of SPECT acquisition. Adding pharmacological data to the Extended model did not result in a notable increase in predictive performance. The AUC increased slightly from .81 to .82, while all other measures remained identical.

## Discussion

### Main findings

We have demonstrated the feasibility of incorporating neuroimaging data, in the form of resting-state rCBF measurements, in the prediction of recidivism in a long-term follow-up of forensic psychiatric patients. The Extended model, which included traditional risk factors as well as resting-state rCBF measurements, saw a 17% increase in AUC, over 200% increase in scaled Brier score, and a 28% increase in accuracy from the Baseline model, which included traditional risk factors only. Recidivists did not significantly differ from non-recidivists in number of patients still under forensic psychiatric care at the end of follow-up, average length of stay, primary diagnosis or time at risk, suggesting that the increased performance of the Extended model was not attributable to any of these variables. Furthermore, supplementary analysis showed that the increased performance in the Extended model was most likely not a result of differences in pharmacological treatment between recidivists and non-recidivists. To the best of our knowledge, this is the first study to include neuroimaging data in the prediction of recidivism in a forensic psychiatric sample, and our results call for continued studies that use neuroimaging methods that potentially could be beneficial also in current clinical forensic practice.

### Baseline model: Traditional risk factors

A cluster B personality disorder, age at first crime and age at FPI emerged as important in both models, similar to previously published findings from the full UPPRÄTT-Malmö cohort [45]. Partial dependence plots revealed that younger age at first crime as well as younger age at FPI increased the probability of being classified as a recidivist in both the Baseline and the Extended model. Furthermore, the effect of age at first crime was larger than the effect of age at FPI. These results are in line with the long-standing observation that *early-onset* aggressive and antisocial behavior increases the risk of life-course persistent criminality [1,10,80]. Likewise, a cluster B personality disorder increased the probability of being classified as a recidivist, in agreement with previous literature showing an increased risk of criminal and violent behavior in individuals who have received diagnoses within this cluster of personality disorders [81,82]. We, as many others before us [83], therefore recommend sustained efforts to identify and support individuals exhibiting antisocial tendencies at a young age.

Substance use disorder was a moderately important predictor of increased probability of recidivism in the Baseline model, although the effect was diminished in the Extended model. Recent research has demonstrated several unfavourable long-term outcomes of substance use in adolescents and young adults with mental disorders [84], and the combination of substance use and mental disorders seems to confer a higher risk of recidivism than substance use or mental disorder alone [85]. Unfortunately, treatment of substance use disorders in mentally disordered offenders appears rare [86,87], even though it may have a positive impact on reducing recidivism.

Several of the traditional risk factors showed no appreciable effect on the prediction of recidivism. Psychopathy, for instance, was not an important predictor in terms of mean decrease in accuracy, although the partial dependence plot revealed that higher PCL:SV scores tended to confer a higher probability of recidivism in the Baseline model. In our study, recidivists scored an average 12 points on the PCL:SV, which is relatively low when contrasted with suggested cutoffs at ≥ 18 for psychopathy and 13–17 for possible psychopathy [88]. It is possible that the low scores and low variability between recidivists and non-recidivists were not sufficient for psychopathic traits to be considered an important variable. It is also possible that using the separate Part 1 (i.e., interpersonal and affective features) and Part 2 (i.e., unstable and antisocial lifestyle) of the PCL:SV would have led to a greater predictive effect of psychopathic traits. Still, research has suggested that both the PCL:SV total score, Part 1 score, and Part 2 score are similar in their ability to predict recidivism [89] as well as violence and aggression [90,91]. In addition, five PCL:SV ratings were performed retrospectively based on file reviews only, which also may have influenced our results, although research has shown that retrospective, file-only PCL:SV ratings can be used reliably for research purposes in Swedish forensic psychiatric populations [63]. Previous criminality was not associated with an increased probability of recidivism, which was a surprising find. However, since previous criminality was operationalized as any convictions *prior* to the crime that lead to FPI and subsequent forensic psychiatric care, all participants, by definition, had already committed a crime when the follow-up started. In other words, a “criminal history” already existed for each patient in our study, which may have diluted the effects of the previous criminality variable (i.e., a possible ceiling effect). We found no effect of mental disorder in a first-degree relative, even though early psychosocial adversities such as parental abuse, parental absence and parental mental disorder have been associated with several negative outcomes, including criminality [7,92]. However, there is evidence of a dose-response relationship between childhood adversities and negative adult outcomes [93–95], and it may be that mental disorder in a first-degree relative alone was not sufficient to yield a predictive effect. Furthermore, at least one study found that while criminal conviction was linked to parental mental disorder, *multiple* (three or more) convictions was not related to parental mental disorder alone, but to rather to parental criminality, or a combination of both [96], again hinting at a possible ceiling effect. Since only five females were included in the study, the lack of effect of sex is difficult to evaluate. It is worth noting, still, that of the two females that did recidivate, neither committed violent crimes. Finally, no effect of education was found. Meta-analytic results have shown that when separated from employment, education is no longer predictive of general recidivism in mentally disordered offenders, in line with our results [14].

### Extended model: Neuroimaging risk factors

Right parietal lobe rCBF emerged as the most important variable, while left parietal lobe rCBF was the fourth most important variable in predicting recidivism in the Extended model. Recidivists had lower rCBF in both the right and left parietal lobe compared to non-recidivists, and partial dependence plots further revealed that lower bilateral parietal rCBF was associated with an increased probability of being classified as a recidivist. Lower right cerebellar rCBF was a modest predictor of recidivism, as was increased bilateral temporal lobe rCBF.

In agreement with prior studies reporting either reduced parietal lobe glucose metabolism or reduced parietal lobe rCBF in violent, impulsive and aggressive samples [32–35], our results suggest that reduced bilateral parietal rCBF may be an important predictor of general recidivism in forensic psychiatric patients. We theorize that parietal lobe contributions to inhibitory control may function as a pathway to criminal behavior. Specifically, although both frontal and parietal regions are involved in response inhibition [97–100], research has suggested that age-related changes in neural circuitry from childhood to early adulthood results in increased recruitment of parietal and occipital regions in response inhibition, while prefrontal recruitment decreases [101]. Poor response inhibition is a consistent marker of externalizing psychopathology, including alcohol and substance abuse [102–104], ADHD [105], aggression [106], as well as violent [107] and non-violent [108] criminality, and appears to be primarily genetic in origin [109]. Thus, an interesting albeit speculative interpretation of our results, given previous evidence of parietal involvement in response inhibition, is that reduced parietal rCBF may lead to poorer inhibitory control, subsequently increasing the risk of recidivism. Speaking against our proposal of parietal lobe contributions to reduced inhibitory control as a pathway to criminal behavior in the current study, however, is the fact that no apparent predictive effect of the frontal lobes was found, despite that frontal regions are robustly activated during response inhibition [100]. In addition, since no test-based measure of behavioral inhibitory control was available, our proposal remains speculative. Future research should further explore possible relationships between parietal lobe function, inhibitory control and criminal behavior.

The role of the cerebellum in criminal and antisocial behavior is relatively unexplored. The cerebellum is known to be recruited during a wide range of cognitive tasks [110], and previous research has demonstrated that cerebellar lesions can lead to a range of psychopathologies, including disinhibited behaviors such as impulsivity and poor attention [111]. Thus, it is possible that reduced cerebellar blood flow leads to an increased risk of disinhibited behavior, similar to what is observed in cerebellar cognitive affective syndrome [112,113]. Recent studies have found that cerebellar gray and white matter volume may be related to criminality, anger and psychopathic traits [30,42], and while our study adds to previous research suggesting cerebellar involvement in criminal behavior, reconciling our results with studies of cerebellar gray and white matter volume it not straightforward, and more research is clearly needed. Using functional magnetic resonance imaging, for instance, it should be possible to study the association between rCBF, GMV and criminal, antisocial or disinhibited behavior.

Our results seem to be at odds with previous reports of frontal and temporal lobe aberrations related to antisocial behavior. Several meta-analytic or review studies have concluded that reduced frontal lobe structure and function appears to be a consistent neurobiological marker of antisocial behavior [25–28]. Still, authors have noted that observed effects are modest [28], not sufficient to cause physical aggression or violence on their own [26], and localized to smaller subregions [25]. Since rCBF was averaged across the left and right frontal lobe in our study, possible effects of rCBF in smaller subregions may have been diffused, a problem that may be resolved using neuroimaging techniques with higher spatial resolution. It is also possible that the entire sample exhibited reduced frontal rCBF, although with little variability between recidivists and non-recidivists. Unfortunately, the lack of a suitable comparison group makes it impossible to investigate this theory. Finally, the negative association between age at FPI and left frontal rCBF is in line with previous research, although it remains unclear why a significant association was not found for the right frontal lobe, as previous findings suggest bilateral reductions [59,61].

Several studies have reported decreased temporal lobe function in aggressive and antisocial samples [35,114,115], although at least one study found no reductions in a sample of violent offenders [32]. In the present study, increased temporal lobe rCBF provided a modest increase in the probability of being classified as a recidivist. Recent studies have revealed a positive association between threat-related amygdala response and impulsive aggression [116–118], and there is prior evidence of perceived threat mediating the relationship between psychosis proneness and aggressive behavior [119] suggesting that increased activity in at least one small temporal lobe subregion may be related to some forms of antisocial behavior. Since our study used a resting-state paradigm, however, no threat-related responses were expected. In addition, the observed effect was modest and thus should be interpreted with caution.

A final consideration is that a majority of the patients (68%) in the current study had a primary diagnosis of psychotic disorder. Psychotic disorders are characterized both by functional aberrations [120] and progressive gray matter reductions [121–123], with some reductions even attributable to pharmacological treatment [124]. The complex nature of brain changes in the various stages of psychotic disorders makes it difficult to compare our results with, for instance, studies of neurobiological contributions to recidivism in non-psychotic populations.

### Strengths and limitations

This study has some notable strengths and several limitations. The average time at risk was ten years, which is rare in forensic psychiatric samples and even rarer when combined with neuroimaging. We included data on pharmacological treatment, which is often overlooked in psychiatric samples, and provided a detailed clinical description of the sample. We employed modern statistical techniques, and all analysis code is publicly available.

As for limitations, a general problem when interpreting results from forensic investigations is that very heterogeneous samples have been studied. For instance, the participants in the current study varied in age, sex, diagnoses, and type of crimes committed. Also, even though we had access to the psychiatric diagnoses that were assessed concurrently with the SPECT investigation, we did not have any data on specific psychiatric symptoms, such as level of psychotic symptoms, at the time of SPECT. In addition, the small sample size means that our results must be carefully interpreted, and readers should refrain from drawing firm conclusions regarding the neurobiological contributions to recidivism before our results are replicated in independent, larger samples. While we included data on pharmacological treatment, we have no information about actual adherence, although adherence is generally believed to be high at forensic psychiatric units due to the possibilities of control of medication intake. Another limitation is seen in our subjective choice of baseline risk factors, which although guided by previous research may have affected predictive accuracy and thus the incremental effect of neuroimaging data. For instance, we did not assess the effect of IQ, which has been associated with CBF in both children and adolescents [125] and in adults [126]. The clinical status of many of the included patients made it difficult to perform reliable and valid IQ assessments at the time of FPI, and these assessments were thus omitted from the protocol. Furthermore, predicting measures other than recidivism could have rendered different results. Future research may compare models predicting recidivism with models predicting other outcome measures, such as the number of adverse incidents during in-patient care and including measures of self-reported criminality. Future studies may also benefit from the complementary information gained from survival models that estimate how the probability of recidivism increases over. We opted not to include such models in the current study due to the limited sample size and relatively large number of predictors used, which limits the interpretability of our results. Finally, the low spatial resolution of the SPECT methodology used prevented detailed study of smaller neural subregions of interest, such as the anterior cingulate cortex [43,44] or the angular gyrus [32,35]. Since it is possible that only smaller subregions of the ROIs included in the current study may be linked to recidivism, using large ROIs may lead to oversimplification and reduced power [127].

### Implications for clinical practice and directions for future research

We have demonstrated that improvements in recidivism prediction in forensic psychiatry may be achievable if neuroimaging data is incorporated into risk assessment models. We reiterate, however, that the study is exploratory in nature, that the results should be carefully interpreted, and that further studies are needed before generalizations can be made. Importantly, the number needed to detain (NND) [128], based on the observed recidivism rate of 36% during our follow-up was 2 in the Baseline model and 1.4 in the Extended model, which is of doubtful clinical relevance. However, the NND depends on the rate of recidivism. Thus, if we assume a recidivism rate of 10% (a plausible rate for a shorter time span, such as one year) the Extended model would reduce the NND by 50%, from 6 in the Baseline model to 3. In addition, the large increase in scaled Brier score in the Extended model suggests an improvement in predictive performance that is not obvious by looking solely at measures based on confusion matrices. Since discriminatory performance metrics such as AUC, accuracy, and NND require predicted probabilities to be binary, valuable information is lost. For instance, if a correct prediction is 0, using a threshold of 0.5, a prediction of 0.1 and a prediction of 0.49 are weighted equally; both will be regarded as 0, even though the former is obviously closer to the truth. Brier scores, on the other hand, utilize all information available in the predicted probabilities. The large increase in scaled Brier score in the Extended model indicates that while the difference in NND may not be clinically relevant, the improved predictive performance of the Extended model may still be useful. For instance, a possible clinical application of improved prediction models would be as decision support systems, aiding physicians in directing resources and interventions to patients at the highest risk of recidivism [129]. A patient with a predicted probability of 0.49 thus should have higher priority in risk management than a patient with a predicted probability of 0.1, even though both fall under the (arbitrary) threshold of 0.5.

Finally, since SPECT measurements are unlikely to be available in most risk assessment situations, this further limits clinical application of our results. However, the primary purpose of this study was to examine if incorporating neuroimaging data leads to incremental increase in predictive performance, and we have shown that using measures of rCBF, an incremental increase in predictive performance is possible. We urge researchers to further assess incremental increases in predictive performance using other neuroimaging techniques, such as functional magnetic resonance imaging. In addition, electroencephalography, while not strictly a neuroimaging method, may be more feasible in clinical settings.

In conclusion, forensic psychiatry is uniquely positioned at the intersection between neuroscience and law. Recently, the emerging field of neurolaw has brought to light the many ethical challenges and questions that unfold in this intersection [19]. Notable examples include reductionism and stigmatization of individuals based on brain function, which are–and have been historically–important ethical challenges that must be addressed with care [24]. Neuroscientific data must be applied responsibly in any context, and if employed in real life legal scenarios, multiple measurement techniques and multiple cognitive tasks should be used [23,24]. Thus, future research should assess the validity of our results in larger, carefully characterized samples, utilizing different and preferably multiple neuroimaging techniques along with assessments of psychiatric symptoms and pharmacological treatment at the time of imaging, and finally test prediction models in held-out samples. Furthermore, our findings and subsequent discussion regarding rCBF measurements and the prediction of recidivism should be interpreted as preliminary, indicating possible avenues for further research rather than definite neural correlates of criminality. The nature of antisocial and criminal behavior is complex and dynamic, and measurements of blood flow in a single particular region of the brain likely only accounts for a small amount of variance in criminal behavior.
